# Impact of high sodium intake on stomach cancer burden in China: A comprehensive analysis from 1990 to 2021

**DOI:** 10.1371/journal.pone.0334593

**Published:** 2026-01-05

**Authors:** Zhouwei Zhan, Yi Zeng, Rui Huang, Jiami Yu, Hui Lin, Xiaojie Wang, Zengqing Guo, Bijuan Chen

**Affiliations:** 1 Department of Medical Oncology, Clinical Oncology School of Fujian Medical University, Fujian Cancer Hospital, Fuzhou, Fujian, China; 2 Department of Gastric Surgical Oncology, Clinical Oncology School of Fujian Medical University, Fujian Cancer Hospital, Fuzhou, Fujian, China; 3 Digestive Endoscopy Center, Clinical Oncology School of Fujian Medical University, Fujian Cancer Hospital, Fuzhou, Fujian, China; 4 Department of Radiation Oncology, Clinical Oncology School of Fujian Medical University, Fujian Cancer Hospital, Fuzhou, Fujian, China; Nazarbayev University School of Medicine, KAZAKHSTAN

## Abstract

**Background:**

Stomach cancer remains a significant public health concern in China, with dietary factors, particularly high sodium intake, being major contributors. This study aimed to quantify the trends, demographic disparities, and contributing factors to this burden from 1990 to 2021.

**Methods:**

Data from the Global Burden of Disease Study (GBD) 2021 were used to assess the burden of stomach cancer attributable to high sodium intake. The study included age-standardized mortality rates (ASMR), disability-adjusted life years (DALYs), years lived with disability (YLDs), and years of life lost (YLLs). Joinpoint regression and age-period-cohort (APC) analysis were performed to identify significant trends and changes. Decomposition analysis was used to identify the impacts of aging, epidemiological changes, and population growth.

**Results:**

In 2021, stomach cancer attributable to high sodium intake resulted in 36,958 deaths in China, with males (26,171) being more affected than females (10,786). The ASMR was significantly higher in males (2.71 per 100,000) than in females (0.99 per 100,000). DALYs totaled 883,435, with males contributing 643,008 and females 240,427. Age-standardized DALY rates were 62.16 for males and 22.15 for females. The age-standardized rates for YLDs and YLLs were also higher in males than females. Although ASMR and age-standardized DALYs rates declined overall (AAPC: −2.45% and −2.76%, respectively), absolute burden increased due to aging and population growth. Compared to global averages, China’s age-standardized rates remained higher, despite notable improvements since 1990. APC analysis showed elevated risks in older age groups, declining period effects, and lower burden among recent birth cohorts. Decomposition analysis indicated that aging and population growth contributed to increased deaths, while epidemiological changes led to a reduction.

**Conclusions:**

Despite declining age-standardized rates, the absolute burden of stomach cancer linked to high sodium remains substantial in China. Males and older adults are disproportionately affected. Population aging and growth are key contributors, while public health interventions have mitigated the burden to some extent. Sustained, gender-sensitive strategies focused on sodium reduction are essential to further lower disease impact.

## Introduction

Stomach cancer, also known as gastric cancer, continues to represent a major global health burden, ranking fifth in incidence and fourth in cancer-related mortality worldwide [1]. The burden of stomach cancer is particularly pronounced in East Asian countries, including China, where dietary and lifestyle factors play a significant role in disease etiology [2–4]. Among modifiable risk factors, high sodium intake has garnered growing attention due to its established role in gastric mucosal damage, promotion of Helicobacter pylori colonization, and facilitation of carcinogenic processes [5–7]. Mechanistically, excessive salt intake has been shown to disrupt the gastric epithelium, impair mucosal barriers, and enhance inflammatory responses, all of which contribute to an increased risk of gastric carcinogenesis [8]. In this context, two recent studies are particularly relevant: a China-focused analysis using Global Burden of Disease (GBD) Study 2019 data through 2019 that documented geographic, sex, and age disparities in sodium-attributable gastric cancer burden [9], and a cross-national study using GBD 2021 that compared China, Japan, and South Korea through 2021 and projected trends to 2042 [10]. Together, these works underscore the public health importance of sodium reduction while highlighting the need for China-specific, up-to-date assessments and deeper exploration of temporal drivers.

Previous meta-analyses and pooled cohort studies have consistently demonstrated a positive dose-response relationship between dietary sodium and stomach cancer risk [11,12]. This association is further modulated by co-exposure to H. pylori and genetic susceptibility, especially among East Asian populations [13]. In China, traditional dietary patterns involving frequent consumption of preserved vegetables, pickled foods, and salted meats elevate the average sodium intake far above World Health Organization recommendations [14,15]. Although national salt-reduction initiatives have been implemented, high-sodium eating habits—particularly in rural communities and older adults—remain prevalent. The China-specific GBD 2019 study provided a valuable baseline up to 2019 [9], while the cross-national GBD 2021 analysis situated China within a broader East Asian context and emphasized forecasting [10]. However, neither work offered a China-focused decomposition of demographic versus epidemiologic drivers alongside formal age-period-cohort modeling and statistically dated inflection points, which are essential to translate surveillance into targeted prevention policy.

To address this gap, the present study provides a comprehensive assessment of the burden of stomach cancer attributable to high sodium intake in China from 1990 to 2021, utilizing data from the GBD Study 2021 [16,17]. We extend prior GBD-based evidence by pairing updated Chinese estimates through 2021 with three complementary analytical lenses: joinpoint regression to identify temporal inflection points, age-period-cohort models to disentangle age, period, and cohort effects, and Das Gupta decomposition to quantify contributions of population aging, population growth, and epidemiologic change. We also contrast China’s age-standardized indicators with global patterns to contextualize national progress. By integrating these methods, our study updates and deepens the narrative established by earlier China-focused and cross-national reports [9,10], providing actionable evidence to guide sodium reduction strategies and gastric cancer control among high-risk demographic groups in China.

## Methods

### Data sources

This study is based on data from the GBD 2021, a comprehensive epidemiological dataset that systematically estimates disease burden across 204 countries and territories from 1990 to 2021. The GBD database provides annual estimates for incidence, prevalence, mortality, DALYs, YLDs, and YLLs, disaggregated by age, sex, location, and cause. Specifically, we extracted estimates of stomach cancer burden attributable to a diet high in sodium for China, covering the period from 1990 to 2021. The attributable burden was determined using the comparative risk assessment framework, which calculates the population attributable fraction based on exposure levels and relative risks obtained from meta-analyses and cohort studies [16,18]. Exposure data for dietary sodium intake were derived from multiple sources, including national nutrition surveys, household dietary data, and food balance sheets, with adjustments for measurement error and representativeness using Bayesian meta-regression models. Disease outcomes and cause-specific mortality estimates were based on data from cancer registries, vital registration systems, verbal autopsy studies, and surveillance reports, harmonized using standardized algorithms in the GBD analytical pipeline. All GBD estimates are accompanied by 95% uncertainty intervals (UIs), which reflect variability due to input data and modeling approaches. The data used in this analysis are publicly available via the Global Health Data Exchange platform (http://ghdx.healthdata.org).

### Definition and estimation

In this study, we estimated the burden of stomach cancer attributable to a diet high in sodium using the comparative risk assessment framework established by the GBD 2021. A diet high in sodium was defined as average 24-hour urinary sodium excretion greater than 1–5 grams per day, based on evidence linking elevated sodium intake with increased risk of gastrointestinal malignancies [16,18]. Stomach cancer was defined as malignant neoplasms of the stomach, corresponding to ICD-10 code C16, and includes all histologic subtypes and tumor locations within the stomach. The population-attributable fraction (PAF) was calculated using the distribution of sodium exposure and the relative risks (RRs) of vstomach cancer across exposure levels. RRs were derived from high-quality cohort studies and meta-analyses examining the relationship between sodium intake and stomach cancer incidence. The theoretical minimum risk exposure level (TMREL) for sodium was determined based on epidemiologic evidence as the level associated with the lowest disease risk. The estimated PAF was applied to the total number of deaths, YLLs, YLDs, and DALYs from stomach cancer to derive the burden attributable to high sodium intake for each age-sex-year-location stratum. All estimates were generated through the GBD modeling platform using the DisMod-MR 2.1 Bayesian meta-regression tool, which integrates input data from dietary surveys, biomarker studies, and administrative health records. Uncertainty was quantified using 1,000 posterior simulations, with final results presented as point estimates and corresponding 95% UIs.

### Descriptive analysis

We first described the burden of stomach cancer attributable to high sodium intake in China by examining deaths, DALYs, YLDs, and YLLs for the year 2021 across sexes and age groups. The absolute numbers and age-standardized rates (per 100,000 population) were presented to characterize the current burden. Age-standardization was performed using the GBD world standard population to allow comparability across years and demographic groups. The distributions of burden across different age groups and by sex were visualized using histograms and line graphs to illustrate disparities and identify peak burden age ranges. Temporal trends from 1990 to 2021 in the absolute numbers and age-standardized rates of deaths, DALYs, YLDs, and YLLs were also analyzed to capture the dynamic changes in disease burden over time. Sex-specific and age-specific comparisons between 1990 and 2021 were performed to assess shifts in burden patterns, particularly focusing on differences in middle-aged and elderly populations. Global comparisons were made by contrasting the Chinese trends with global averages for the same period, enabling evaluation of China’s relative progress in stomach cancer prevention attributable to sodium reduction. All descriptive statistics were reported as means with corresponding 95% UIs to account for sampling and model uncertainty inherent in GBD estimates.

### Joinpoint regression analysis

Joinpoint regression analysis was employed to identify significant changes in the temporal trends of stomach cancer burden attributable to high sodium intake in China from 1990 to 2021. This method enables the detection of inflection points (joinpoints) where trends in age-standardized rates significantly change direction or magnitude, providing a more nuanced understanding of the time-series data. The analysis was applied to age-standardized rates of deaths, DALYs, YLDs, and YLLs for both sexes combined, as well as separately for males and females. Annual percent change was calculated for each segment between identified joinpoints, and the average annual percent change (AAPC) was computed for the overall study period. A Monte Carlo permutation method was used to determine the number of joinpoints and test the statistical significance of each trend segment [19,20]. A *p*-value of less than 0.05 was considered statistically significant. All estimates were reported with 95% confidence intervals (CIs). The Joinpoint Regression Program (version 5.2.0) developed by the U.S. National Cancer Institute was used for all analyses. This approach allowed us to quantify the timing and magnitude of changes in burden over time and assess whether trends were consistent across demographic subgroups.

### Age-period-cohort (APC) analysis

To assess the independent effects of age, time period, and birth cohort on trends in stomach cancer burden attributable to high sodium intake in China, an APC model was applied. This analytical approach enables the separation of temporal patterns into three distinct dimensions, thus providing deeper insight into how demographic aging, historical context, and generational exposures influence the disease burden. Due to the inherent collinearity among age, period, and cohort variables, the intrinsic estimator (IE) method was employed to generate unbiased and statistically robust estimates of each effect [21,22]. Data on mortality and DALYs due to stomach cancer from 1990 to 2021 were extracted from the GBD 2021 database. These data were organized into consecutive five-year age groups ranging from 25–29–95 + , and corresponding five-year time intervals (e.g., 1992–1996, 1997–2001). Individuals younger than 25 and older than 95 were grouped into terminal categories to ensure analytical consistency. Cumulative age-specific and period-specific rates were calculated accordingly. The APC model was implemented using the Epi package (version 2.46) in R (version 4.3.1). Model fit was evaluated based on residual plots and Akaike Information Criterion (AIC) values to ensure the best representation of temporal trends.

### Decomposition analysis

To quantify the relative contributions of population aging, population growth, and epidemiological change to the observed variation in stomach cancer burden attributable to high sodium intake in China between 1990 and 2021, a decomposition analysis was conducted. This method isolates the effect of demographic and epidemiologic drivers on changes in the number of deaths and DALYs over time, enabling a clearer understanding of the underlying forces shaping the disease burden. We applied the decomposition method based on the Das Gupta approach [23,24], which disaggregates the absolute change in disease burden into three additive components: (1) changes due to population growth, reflecting the increase in total population size; (2) changes due to population aging, reflecting the shift in age distribution toward older age groups; and (3) changes due to epidemiological factors, including variations in age-specific disease rates due to prevention, treatment, or behavioral changes. Each component was estimated while holding the other two constant, thereby allowing an independent evaluation of its impact on the net change in burden. This analysis was stratified by sex to assess gender-specific drivers and was conducted separately for both deaths and DALYs. Results were visualized to show the directional effect (positive or negative) of each component across the two outcomes.

### Ethical considerations

This study utilized publicly available data from the GBD Study, which does not contain any individual-level identifiers. We adhered to the GATHER statement throughout the research process to ensure that the handling and reporting of the data were in line with ethical and scientific best practices [25].

## Results

### Burden of stomach cancer attributable to high sodium intake in China, 2021

In 2021, the burden of stomach cancer attributable to a diet high in sodium remained a significant public health concern in China. The total number of deaths attributable to high sodium intake reached 36,958 cases, with a marked gender disparity: 26,171 deaths among males and 10,786 among females. Correspondingly, the age-standardized death rate was considerably higher in males (2.71 per 100,000) than in females (0.99 per 100,000), with an overall rate of 1.78 per 100,000 population. DALYs, reflecting the combined impact of premature mortality and morbidity, totaled 883,435 for both sexes, with 643,008 in males and 240,427 in females. The age-standardized DALYs rate was 62.16 per 100,000 in males and 22.15 per 100,000 in females, averaging 41.46 overall. Most of this burden stemmed from YLLs, which accounted for 870,108 of the total DALYs, while YLDs contributed a smaller portion at 13,327. The age-standardized rates for YLLs and YLDs also showed pronounced sex differences, further emphasizing the disproportionate impact of high sodium consumption on male populations (Table 1).

**Table 1:** All-age cases and age-standardized deaths, DALYs, YLDs, and YLLs rates in 2021 for stomach cancer attributable to diet high in sodium in China.

Measure	All-ages cases			Age-standardized rates per 100,000 people		
	Total	Male	Female	Total	Male	Female
Deaths	36958 (0, 183972)	26171 (0, 136689)	10786 (0, 55030)	1.78 (0, 8.81)	2.71 (0, 14.07)	0.99 (0, 5.06)
DALYs	883435 (0, 4461211)	643008 (0, 3367599)	240427 (0, 1225234)	41.46 (0, 208.59)	62.16 (0, 323.76)	22.15 (0, 2.91)
YLDs	13327 (0, 68749)	9932 (0, 52416)	3395 (0, 17570)	0.63 (0, 3.23)	0.97 (0, 5.1)	0.31 (0, 1.62)
YLLs	870108 (0, 4405065)	633076 (0, 3318215)	237032 (0, 1209225)	40.83 (0, 205.96)	61.2 (0, 319.63)	21.84 (0, 1.45)

Values in parentheses indicate 95% UIs, estimated using Monte Carlo simulations. Abbreviations: DALYs, disability-adjusted life-years; YLDs, years lived with disability; YLLs, years of life lost.

### Age and sex patterns of stomach cancer burden attributable to high sodium intake in China, 2021

**Fig 1:**
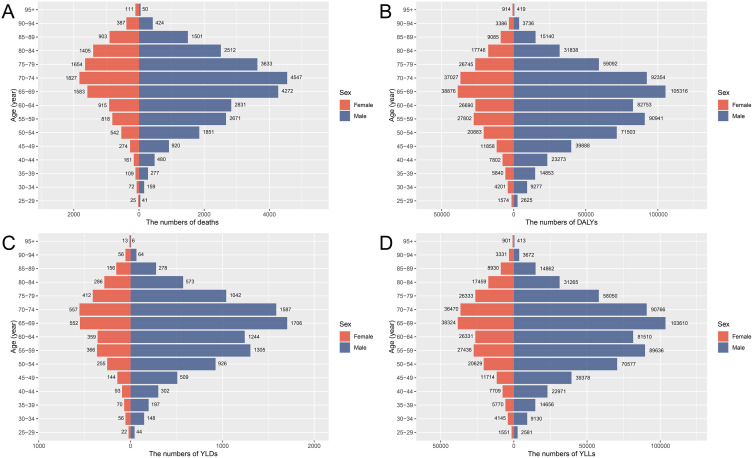
The burden of stomach cancer attributable to high sodium intake in China by sex and age group in 2021. (A) Number of deaths by sex and age group. (B) Number of DALYs by sex and age group. (C) Number of YLDs by sex and age group. (D) Number of YLLs by sex and age group. Values in parentheses indicate 95% UIs, estimated using Monte Carlo simulations. Abbreviations: DALYs, disability-adjusted life years; YLDs, years lived with disability; YLLs, years of life lost.

**Fig 2:**
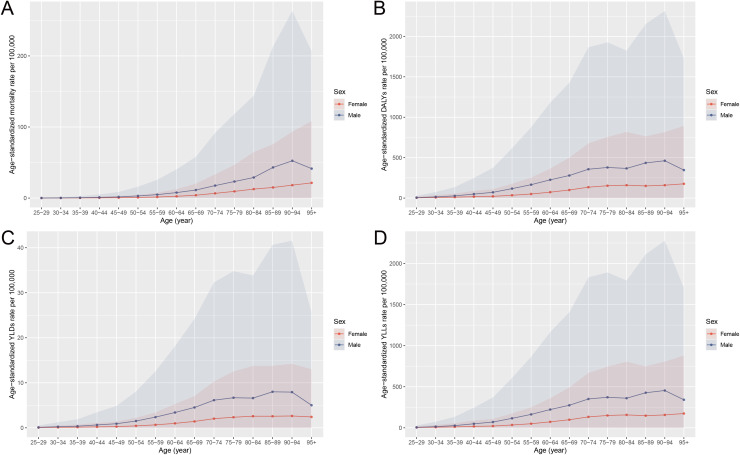
Age-standardized rates of stomach cancer burden attributable to high sodium intake in China by sex and age group in 2021. (A) Age-standardized mortality rates per 100,000 population. (B) Age-standardized DALYs rates per 100,000 population. (C) Age-standardized YLDs rates per 100,000 population. (D) Age-standardized YLLs rates per 100,000 population. Abbreviations: DALYs, disability-adjusted life years; YLDs, years lived with disability; YLLs, years of life lost.

In 2021, the distribution of stomach cancer burden by age and sex reveals pronounced disparities. As shown in Fig 1, males experience a consistently higher number of deaths, DALYs, YLDs, and YLLs across all age groups compared to females, with the highest burden observed in the 65–74 age range. Among males, both deaths and DALYs peak within this age group, and the YLLs reach their highest point as well, indicating a substantial loss of life years due to premature mortality. Although females show a lower overall burden, they exhibit similar age-related trends, with peak values occurring in the same age range. Fig 2 illustrates the corresponding age-specific rates for these indicators. Mortality, DALYs, YLDs, and YLLs rates all increase with age, with a marked rise beginning around the 55–60 age group for both sexes. Across all age groups, males exhibit higher rates than females, with a particularly steep increase seen in men, underscoring the greater vulnerability of males to high sodium-associated gastric cancer. These patterns highlight the urgent need for targeted sodium reduction interventions, especially among middle-aged and older men. S1 Fig further emphasizes the differential impact of risk factors by gender. In females, high sodium intake accounts for the majority of stomach cancer deaths and DALYs (81.3 percent and 82.8 percent, respectively), whereas in males, smoking is the predominant contributor, responsible for 70.7 percent of deaths and 70.0 percent of DALYs.

### Temporal and age-specific trends in stomach cancer burden attributable to high sodium intake in China, 1990–2021

**Fig 3:**
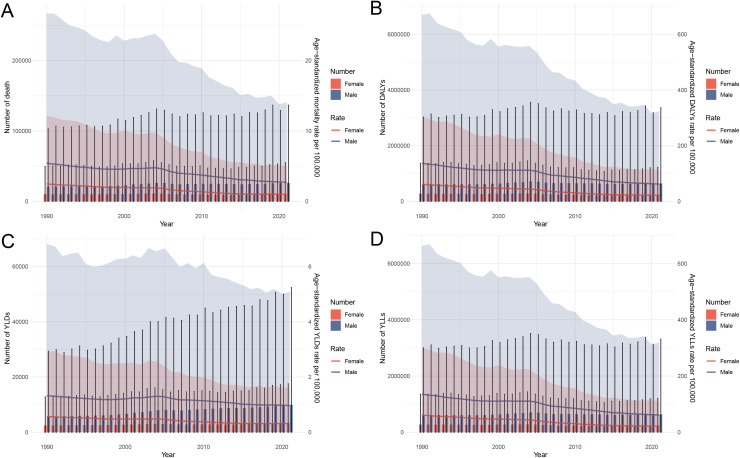
Temporal trends in the burden of stomach cancer attributable to high sodium intake in China from 1990 to 2021, by sex. (A) Number and age-standardized mortality rate per 100,000 population. (B) Number and age-standardized DALYs rate per 100,000 population. (C) Number and age-standardized YLDs rate per 100,000 population. (D) Number and age-standardized YLLs rate per 100,000 population. Abbreviations: DALYs, disability-adjusted life years; YLDs, years lived with disability; YLLs, years of life lost.

**Fig 4:**
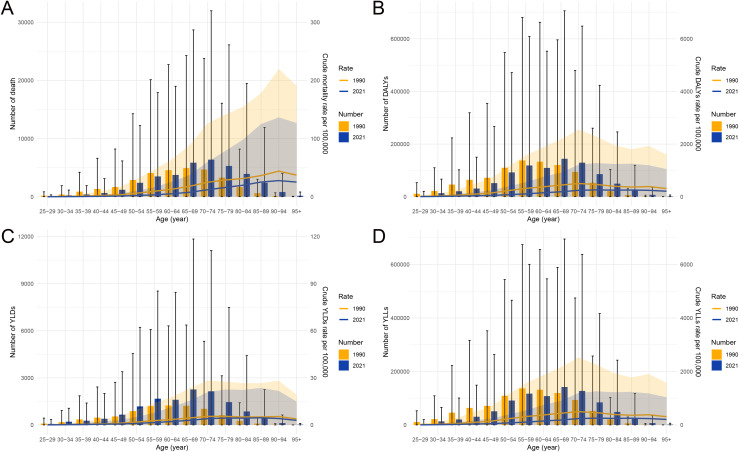
Age-specific comparisons of stomach cancer burden attributable to high sodium intake in China between 1990 and 2021. (A) Number and crude mortality rate per 100,000 population. (B) Number and crude DALYs rate per 100,000 population. (C) Number and crude YLDs rate per 100,000 population. (D) Number and crude YLLs rate per 100,000 population. Abbreviations: DALYs, disability-adjusted life years; YLDs, years lived with disability; YLLs, years of life lost.

From 1990 to 2021, the burden of stomach cancer attributable to high sodium intake in China demonstrated notable changes in both magnitude and distribution across age groups. As shown in Fig 3, the absolute number of deaths, DALYs, YLDs, and YLLs increased over time, particularly among males. Although the age-standardized rates for mortality, DALYs, and YLLs showed a general decline, reflecting improvements in prevention and control, the total burden continued to rise due to population aging and growth. Fig 4 further reveals age-specific patterns by comparing data from 1990 and 2021. In 2021, the number of deaths, DALYs, and YLLs was higher among individuals aged 65 years and older compared to 1990, underscoring the increasing burden in the elderly population. In contrast, the number of YLDs rose beginning in the 45–49 age group, suggesting an expansion of the disease’s disabling impact into younger age brackets. These trends highlight the shifting demographic of disease burden and underscore the importance of age-tailored prevention strategies, particularly sodium reduction efforts targeting middle-aged and elderly populations to curb both mortality and morbidity associated with stomach cancer.

### Comparison of Chinese and global trends in stomach cancer burden attributable to high sodium intake, 1990–2021

**Fig 5:**
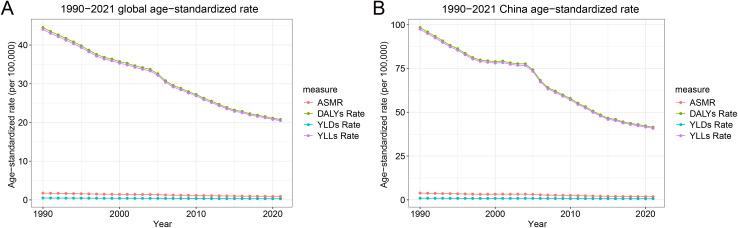
Trends in age-standardized rates of stomach cancer attributed to high sodium intake globally and in China, 1990–2021. (A) Global ASMR, DALYs, YLDs, and YLLs per 100,000 population. (B) Age-standardized rates in China for the same indicators. Abbreviations: ASMR, Age-standardized mortality rate; DALYs, disability-adjusted life years; YLDs, years lived with disability; YLLs, years of life lost.

Between 1990 and 2021, China experienced a notable decline in the age-standardized burden of stomach cancer attributable to high sodium intake, though the absolute burden remains comparatively high. As shown in Table 2 and Fig 5, age-standardized death rates in China decreased from 3.85 to 1.78 per 100,000 population, while the global rate declined from 1.74 to 0.89 per 100,000. Similarly, DALYs rates in China dropped from 98.4 to 41.46, with a global decrease from 44.53 to 20.78 per 100,000. YLDs and YLLs also declined in both settings, with YLLs showing the most substantial decrease, reflecting improvements in premature mortality. Notably, the decline in China’s DALYs and YLLs rates was greater than the global average, with annual percent changes of −2.76 and −2.77, respectively. Despite these improvements, China’s age-standardized rates for all four indicators in 2021 remained higher than global averages, particularly for DALYs and YLLs, underscoring a disproportionately higher burden. These findings highlight the dual impact of effective interventions and the persistent need for intensified sodium reduction efforts in China to further narrow the gap with global trends.

**Table 2:** Change of age-standardized rates in deaths, DALYs, YLDs, and YLLs for stomach cancer attributable to diet high in sodium between 1990 and 2021 in China and global level.

Measure	China			Global		
	1990	2021	Change	1990	2021	Change
Deaths	3.85 (0, 18.79)	1.78 (0, 8.81)	−2.45 (−2.61 - −2.30)^*^	1.74 (0, 8.74)	0.89 (0, 4.37)	−2.17 (−2.30 - −2.05)^*^
DALYs	98.4 (0, 478.5)	41.46 (0, 208.59)	−2.76 (−2.91 - −2.61)^*^	44.53 (0, 222.31)	20.78 (0, 102.38)	−2.45 (−2.56 - −2.34)^*^
YLDs	0.93 (0, 4.73)	0.63 (0, 3.23)	−1.27 (−1.43 - −1.12)^*^	0.48 (0, 2.51)	0.3 (0, 1.57)	−1.56 (−1.70 - −1.43)^*^
YLLs	97.46 (0, 473.84)	40.83 (0, 205.96)	−2.77 (−2.93 - −2.62)^*^	44.05 (0, 219.97)	20.48 (0, 100.84)	−2.46 (−2.57 - −2.35)^*^

Values in parentheses indicate 95% UIs, estimated using Monte Carlo simulations. Abbreviations: DALYs, disability-adjusted life-years; YLDs, years lived with disability; YLLs, years of life lost; ^*^, *p* < 0.05.

### Joinpoint analysis and temporal trends in stomach cancer burden attributable to high sodium intake in China, 1990–2021

**Fig 6:**
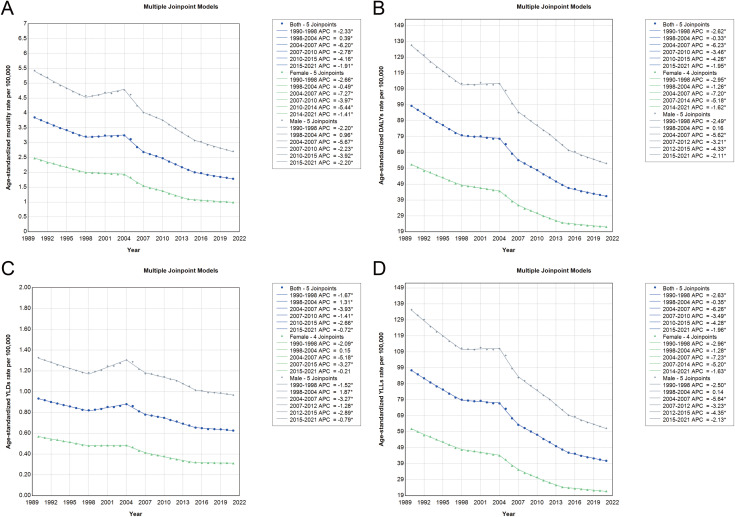
Joinpoint regression analysis of stomach cancer burden attributable to high sodium intake in China from 1990 to 2021. The graph shows trends for both sexes (blue line), females (green line), and males (grey line). (A) ASMR per 100,000 population. (B) Age-standardized DALYs rate per 100,000 population. (C) Age-standardized YLDs rate per 100,000 population. (D) Age-standardized YLLs rate per 100,000 population. Abbreviations: ASMR, Age-standardized mortality rate; DALYs, disability-adjusted life years; YLDs, years lived with disability; YLLs, years of life lost.

Joinpoint regression analysis revealed distinct temporal patterns in the age-standardized rates of stomach cancer burden attributable to high sodium intake in China between 1990 and 2021 (Fig 6, S1 and S2 Tables). Overall, the age-standardized mortality, DALY, YLD, and YLL rates declined significantly over the period, with the sharpest declines occurring between 2004 and 2007 for all four indicators. The average annual percent change (AAPC) for the full period showed consistent decreases: mortality (−2.45%), DALYs (−2.76%), YLDs (−1.27%), and YLLs (−2.77%). Between 1998 and 2004, a temporary plateau or slight increase was observed in mortality and YLD rates, particularly among males, before a renewed decline resumed post-2004. Gender-specific trends showed a more pronounced decline among females compared to males, especially in the earlier years. Notably, the DALY and YLL rates among females decreased at faster rates during 1990–1998 and again between 2004 and 2007. In contrast, the decline in male rates was slower overall, especially for YLDs. These findings underscore the effectiveness of public health efforts to reduce sodium-related cancer burden over the last three decades, while also highlighting the need for sustained, gender-sensitive interventions to address persisting disparities.

### APC effects on stomach cancer mortality and DALYs attributable to high sodium intake in China, 1990–2021

**Fig 7:**
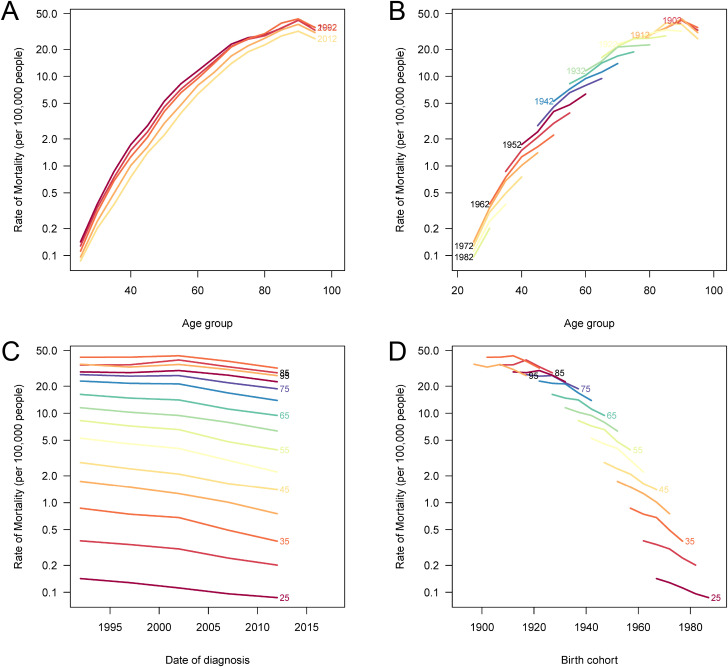
Age-period-cohort analysis of stomach cancer mortality attributable to high sodium intake in China. (A) The age-specific mortality rates according to time periods; each line connects the age-specific rates for a 5-year period. (B) The age-specific mortality rates according to birth cohorts; each line connects the age-specific rates for a 5-year birth cohort. (C) The period-specific mortality rates according to age groups; each line connects the period-specific rates for a 5-year age group. (D) The cohort-specific mortality rates according to age groups; each line connects the cohort-specific rates for a 5-year age group.

The APC analysis revealed significant temporal dynamics in stomach cancer mortality and DALYs attributable to high sodium intake in China (Figs 7 and S2). Age effects showed a clear upward trend, with both mortality and DALYs rates increasing steadily with age, indicating that older individuals bear a substantially higher burden. Period effects demonstrated a gradual decline in mortality and DALYs in more recent time periods, suggesting improvements in medical care and public health interventions over time. Cohort effects further illustrated a consistent downward trend in more recent birth cohorts, reflecting the benefits of improved early-life nutrition, public awareness, and sodium reduction policies implemented over the past decades. Together, these findings highlight that while the aging population continues to experience a heavier burden, progress in disease prevention and control has led to reduced risk among younger generations, underscoring the value of sustained population-level interventions targeting dietary sodium reduction.

### Decomposition of changes in stomach cancer deaths and DALYs attributable to high sodium intake in China, 1990–2021

**Fig 8:**
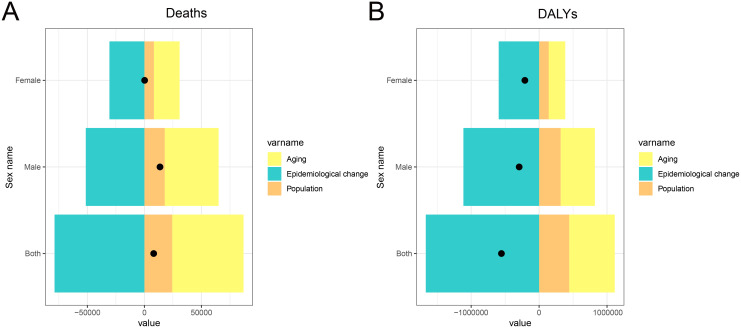
Decomposition analysis of changes in stomach cancer burden attributable to high sodium intake in China from 1990 to 2021. (A) Decomposition of changes in the number of deaths by factor (aging, population growth, epidemiological change), stratified by sex. (B) Decomposition of changes in DALYs by the same factors and sex categories. Abbreviations: DALYs, disability-adjusted life years.

Decomposition analysis identified the key contributors to changes in stomach cancer burden attributable to high sodium intake in China between 1990 and 2021 (Fig 8). The increase in both deaths and DALYs was primarily driven by population aging, with a more pronounced impact observed among males. Population growth also contributed positively to the overall burden across both sexes. In contrast, epidemiological change, marked by advancements in disease prevention, early diagnosis, and clinical management, exerted a mitigating influence, contributing to a partial reduction in the overall burden. These findings underscore that while demographic shifts such as aging and population expansion continue to elevate the disease burden, advancements in health interventions have played a crucial role in mitigating some of these increases, especially in females. The results highlight the need for strengthened prevention strategies targeting modifiable risks, alongside broader health system adaptations to address the growing challenges posed by an aging population.

## Discussion

This study presents a comprehensive evaluation of the long-term burden of stomach cancer attributable to high sodium intake in China from 1990 to 2021, revealing substantial but evolving public health challenges. Although the age-standardized mortality and DALY rates have shown a general decline over the past three decades, the absolute number of deaths, YLLs, and DALYs has increased, primarily due to demographic transitions, including population aging and growth. In 2021, males consistently bore a higher burden than females across all indicators, with the highest impact observed in individuals aged 65 years and older. Joinpoint regression identified key inflection periods, with the steepest declines in burden occurring between 2004 and 2007, suggesting the potential effect of improved healthcare access or policy interventions during that time. The APC analysis indicated a significant increase in risk with age, declining risks across more recent periods, and notably lower risks in younger birth cohorts, implying generational improvements in dietary patterns or public health efforts. Furthermore, decomposition analysis revealed that population aging was the dominant contributor to the rising number of deaths and DALYs, while epidemiological changes had a mitigating effect. These findings highlight both the progress made and the persisting challenges in reducing diet-related stomach cancer in China. The differential burden by sex, age, and birth cohort underscores the need for tailored prevention and control strategies, particularly those targeting older adults and male populations, who remain disproportionately affected despite overall national declines in age-standardized rates.

Our findings align with recent global and national studies showing persistent disparities in the burden of stomach cancer across countries and populations. According to Sharma et al. [26], while the global age-standardized incidence rate and age-standardized mortality rate for stomach cancer have declined between 1990 and 2019, East Asia, especially China, continues to carry the highest burden, with 61.5% of global incident cases and 58.6% of deaths in 2019. This is consistent with our results, which highlight high sodium intake as a prominent risk factor in China, a trend echoed in the GBD-based study by Yang et al. [27], where the DALYs attributable to high sodium diets in China ranked highest among G20 countries. Furthermore, the comparative study by Yang et al. emphasized the persistent gap between China and other G20 nations, suggesting the need for more tailored prevention strategies across demographic groups. While smoking remains a major contributor globally, high sodium diets play a particularly prominent role in East Asian countries, which may be due to regional dietary patterns [28]. Compared with prior reports that focused mainly on smoking and *H. pylori* as major risks [26], our study adds emphasis on sodium-related risks and their temporal trends. Collectively, these findings underscore the importance of adapting global strategies to regional dietary behaviors and reinforce the call for enhanced dietary surveillance and public education campaigns in high-burden regions like China.

High sodium intake promotes stomach carcinogenesis through several interrelated biological mechanisms, many of which have been extensively validated in both experimental and epidemiological studies. One of the key mechanisms involves sodium’s direct injurious effect on the gastric mucosa, which enhances epithelial permeability, triggers persistent inflammation, and promotes proliferation of gastric pit epithelial cells, cumulatively establishing an environment conducive to malignant transformation [8,29]. High sodium consumption also enhances the colonization and virulence of Helicobacter pylori, a known class I carcinogen, by upregulating bacterial virulence genes and amplifying mucosal immune responses, thereby accelerating the progression from gastritis to atrophic gastritis and intestinal metaplasia [29]. In addition to its synergistic interaction with H. pylori, excessive sodium intake facilitates endogenous formation of N-nitroso compounds from dietary nitrates and nitrites, which are potent mutagens implicated in gastric carcinogenesis [30,31]. Moreover, chronic high sodium intake can disrupt the gastric barrier, leading to sustained oxidative stress and cytokine-mediated injury, both of which increase DNA damage and support neoplastic progression [5]. These mechanisms provide strong biological plausibility for the observed associations between high sodium intake and stomach cancer and support the rationale for sodium-reduction strategies as a cancer prevention tool. Given that all Chinese provinces currently exceed WHO-recommended sodium limits, the potential impact of these mechanisms is particularly relevant in the national context [32,33].

The observed gender disparities in the burden of stomach cancer attributable to high sodium intake reflect both biological and behavioral differences between males and females. Across all age groups, males experienced consistently higher rates of deaths, DALYs, YLDs, and YLLs than females, aligning with patterns documented in prior research [4,9]. Men might be more susceptible to high sodium intake’s carcinogenic effects, possibly because of differences in hormone levels and gastric physiology [34,35]. Additionally, higher dietary sodium intake among men, as well as greater exposure to other compounding risk factors such as smoking and alcohol consumption, which synergistically amplify gastric carcinogenesis [36]. Additionally, lower health awareness and reduced access to preventive healthcare services among men may contribute to delayed diagnosis and poorer outcomes. From a demographic standpoint, decomposition analysis indicated that population aging was the principal driver of increased stomach cancer burden, especially among men. This finding is consistent with broader demographic transitions in China, where prolonged life expectancy has led to a growing elderly population that is more vulnerable to chronic diseases [37,38]. In contrast, epidemiological change had a counterbalancing effect, particularly in females, suggesting that improvements in prevention and early diagnosis have been more effective in this group. The APC analysis further highlighted generational differences in disease risk. Younger cohorts exhibited lower mortality and DALYs, indicating a possible benefit from reduced salt preservation in food, the rise of refrigeration, improved hygiene, and health education over recent decades. These shifts may have also contributed to changes in H. pylori infection patterns, dietary composition, and sodium awareness across generations [39,40]. The declining cohort risk underscores the potential long-term benefits of sustained public health initiatives targeting dietary modification and early-life interventions.

The induction of stomach cancer by a high-sodium diet is associated with direct damage to the gastric mucosa, proliferation of gastric pit epithelium, and colonization by H. pylori [8,29]. Sodium restriction effectively reduces stomach cancer incidence and mortality rates. In 2010, the average global sodium intake was 3.95 g per day, with regional intakes ranging from 2.18 to 5.51 g per day [41]. China’s diet is changing, with refrigeration replacing salt for preservation, but high sodium intake persists due to added salt, seasonings, and processed foods. All provinces exceed the recommended daily salt (5 g) and sodium (2 g) limits. Although added salt has decreased, total sodium intake has not, especially in Guangxi. Primary prevention through lifestyle and dietary modifications is crucial to reducing the cancer burden [30]. Further efforts are needed to limit salt/sodium intake, and regular monitoring is needed to assess progress [32,33], with strategies focusing on tailored approaches for rural areas, males, and older individuals. To enhance salt-reduction campaigns in China, national strategies like food reformulation under salt targets and front-of-pack labeling with nutritional information should be considered and integrated into Chinese culture for added effectiveness [42]. Additionally, promoting low-sodium salt and providing salt-related education to the entire population should be strengthened. Given China’s complex environment, multicomponent strategies for salt reduction would be more effective [14].

While this study provides a comprehensive assessment of the stomach cancer burden attributable to high sodium intake in China over the past three decades, several limitations should be acknowledged. First, the estimates were derived from the GBD 2021, which, despite its standardized methodology, relies on secondary data sources that may be limited in coverage, particularly in rural or underrepresented regions. Potential underreporting or misclassification of stomach cancer deaths and dietary sodium exposure could influence the precision of burden estimates [17,43]. Second, sodium intake was estimated using modeled 24-hour urinary sodium excretion rather than direct population-wide biomarker measurements, introducing a degree of uncertainty. Third, a data-currency limitation exists because the most recent GBD cycle used in our analysis extends only to 2021. This lag is inherent to large-scale harmonized datasets and reflects the time required for compilation, validation, and modeling. While stomach cancer indicators typically evolve gradually, unmeasured post-2021 changes (for example, shifts in diet, salt-reduction initiatives, or health service access) are not captured here. To mitigate potential impact for stakeholders, our estimates should be interpreted as a robust baseline for long-term patterns and triangulated with the most recent national surveillance (e.g., updated cancer registry releases and 24-hour urinary sodium monitoring) when informing near-term decisions. We do not anticipate major negative implications provided that programmatic choices consider local contemporaneous data; however, applications requiring real-time evaluation should exercise caution pending newer GBD updates. We have acknowledged this limitation in the Discussion and note our intent to incorporate subsequent GBD releases when available. Additionally, while the comparative risk assessment framework accounts for confounding to some extent, it cannot fully isolate the effects of sodium from other dietary or lifestyle risk factors, such as intake of processed meat, low fruit and vegetable consumption, or concurrent risk exposures like Helicobacter pylori infection and alcohol use [44]. These important contributors to gastric carcinogenesis were not included in the GBD 2021 attributable risk estimates and thus not incorporated into our comparative analysis, which may limit the scope of interpretation. Fourth, cohort effects may be influenced by unmeasured social, economic, or healthcare-related variables that evolved over time. Future research should aim to validate these findings through prospective cohort studies incorporating biomarker-based sodium assessments and stratification by additional known risk factors such as H. pylori status and alcohol consumption patterns. Moreover, studies exploring the synergistic effects of dietary sodium with other risk factors in molecular subtypes of gastric cancer would provide deeper mechanistic insight. Enhanced surveillance and region-specific dietary monitoring are also needed to better inform local interventions. Expanding research into behavioral drivers of high sodium intake could support the design of more effective, culturally appropriate public health campaigns.

## Conclusions

This study provides a comprehensive overview of the long-term burden of stomach cancer attributable to high sodium intake in China, offering critical insights into its temporal dynamics and underlying demographic drivers. The findings underscore the importance of continued efforts to reduce sodium consumption as a cornerstone of cancer prevention and public health improvement. Effective implementation of sodium-reduction strategies, including dietary education, policy regulation, and community-level interventions, remains essential to curbing the future burden. Moreover, addressing disparities across age, sex, and geographic regions will be vital to achieving equitable health outcomes. Future research should integrate individual-level data on sodium intake, Helicobacter pylori infection, and genetic susceptibility to improve risk stratification. Investigations into the molecular mechanisms of sodium-induced gastric carcinogenesis, as well as the combined effects of multiple dietary and lifestyle factors, are warranted to inform more targeted prevention strategies. Multidisciplinary collaborations and enhanced surveillance systems will be key to translating evidence into actionable policy.

## Supporting information

S1 Fig Proportional contribution of risk factors to stomach cancer burden deaths (A-B) and DALYs (C-D) in China, 2021. Abbreviations: DALYs, disability-adjusted life years.
(PDF)

S1 Table Trends in age-standardized mortality and DALY rates (per 100,000 persons) among both sexes, males, and females from 1990 to 2021 for stomach cancer attributable to diet high in sodium in China. Abbreviations: DALYs, disability-adjusted life years.
(DOCX)

S2 Table Trends in age-standardized YLD, and YLL rates (per 100,000 persons) among both sexes, males, and females from 1990 to 2021 for stomach cancer attributable to diet high in sodium in China. Abbreviations: YLD, year lived with disability; YLL, year of life lost.
(DOCX)

S2 Fig Age-period-cohort analysis of DALYs for stomach cancer attributable to high sodium intake in China. (A) The age-specific DALYs rates according to time periods; each line connects the age-specific rates for a 5-year period. (B) The age-specific DALYs rates according to birth cohorts; each line connects the age-specific rates for a 5-year birth cohort. (C) The period-specific DALYs rates according to age groups; each line connects the period-specific rates for a 5-year age group. (D) The cohort-specific DALYs rates according to age groups; each line connects the cohort-specific rates for a 5-year age group. Abbreviations: DALYs, disability-adjusted life years.
(PDF)

## References

[1] Bray F, Laversanne M, Sung H, Ferlay J, Siegel RL, Soerjomataram I, et al. Global cancer statistics 2022: GLOBOCAN estimates of incidence and mortality worldwide for 36 cancers in 185 countries. CA Cancer J Clin. 2024;74(3):229–63. doi: 10.3322/caac.21834 38572751

[2] Thrift AP, Wenker TN, El-Serag HB. Global burden of gastric cancer: epidemiological trends, risk factors, screening and prevention. Nat Rev Clin Oncol. 2023;20(5):338–49. doi: 10.1038/s41571-023-00747-0 36959359

[3] Arnold M, Abnet CC, Neale RE, Vignat J, Giovannucci EL, McGlynn KA, et al. Global Burden of 5 Major Types of Gastrointestinal Cancer. Gastroenterology. 2020;159(1):335-349.e15. doi: 10.1053/j.gastro.2020.02.068 32247694 PMC8630546

[4] Zhan Z, Chen B, Zeng Y, Huang R, Yu J, Guo Z, et al. Long-term trends and projections of stomach cancer burden in China: Insights from the GBD 2021 study. PLoS One. 2025;20(4):e0320751. doi: 10.1371/journal.pone.0320751 40198592 PMC11978042

[5] Balendra V, Amoroso C, Galassi B, Esposto J, Bareggi C, Luu J, et al. High-Salt Diet Exacerbates H. pylori Infection and Increases Gastric Cancer Risks. J Pers Med. 2023;13(9):1325. doi: 10.3390/jpm13091325 37763093 PMC10533117

[6] Silva ARC, Alicandro G, Guandalini VR, da Fonseca Grili PP, Assumpção PP, Barbosa MS, et al. Exploring the link between dietary patterns and gastric adenocarcinoma in Brazil: a mediation analysis. BMC Med. 2024;22(1):562. doi: 10.1186/s12916-024-03785-2 39609810 PMC11603788

[7] Kwak JH, Eun CS, Han DS, Kim HJ. Effects of RAD50 SNP, sodium intake, and H. pylori infection on gastric cancer survival in Korea. Gastric Cancer. 2024;27(2):210–20. doi: 10.1007/s10120-023-01441-x 38070008

[8] Wu X, Chen L, Cheng J, Qian J, Fang Z, Wu J. Effect of Dietary Salt Intake on Risk of Gastric Cancer: A Systematic Review and Meta-Analysis of Case-Control Studies. Nutrients. 2022;14(20):4260. doi: 10.3390/nu14204260 36296944 PMC9609108

[9] Jiang L, Wang A, Yang S, Fang H, Wang Q, Li H, et al. The Burden of Gastric Cancer Attributable to High Sodium Intake: A Longitudinal Study from 1990 to 2019 in China. Nutrients. 2023;15(24):5088. doi: 10.3390/nu15245088 38140347 PMC10745903

[10] Liu W, Peng Z-Z, Zhao D-Q, Liu Y, Liao K. The burden of gastric cancer attributed to high salt intake and predictions through the year 2042: a cross-national comparative analysis of China, Japan, and South Korea. Front Nutr. 2025;12:1584400. doi: 10.3389/fnut.2025.1584400 40697556 PMC12279510

[11] Fu J, Shin W-K, Huang D, De la Torre K, Kang D, Shin S. Fruit and salt consumption are related to the risk of gastric cancer incidence in Asian populations: a comprehensive systematic review and meta-analysis of cohort studies. Epidemiol Rev. 2025;47(1):mxaf007. doi: 10.1093/epirev/mxaf007 40331758

[12] Kronsteiner-Gicevic S, Thompson AS, Gaggl M, Bell W, Cassidy A, Kühn T. Adding salt to food at table as an indicator of gastric cancer risk among adults: a prospective study. Gastric Cancer. 2024;27(4):714–21. doi: 10.1007/s10120-024-01502-9 38630317 PMC11193689

[13] Takasu A, Gotoda T, Suzuki S, Kusano C, Goto C, Ishikawa H, et al. Daily Diet and Nutrition Risk Factors for Gastric Cancer Incidence in a Japanese Population. Gut Liver. 2024;18(4):602–10. doi: 10.5009/gnl230354 38388181 PMC11249943

[14] Zhang W, Neupane D, Zhao Z, Jiang B, Zhang M, Zhang X, et al. Knowledge and practices related to salt consumption in China: findings from a national representative cross-sectional survey. J Hum Hypertens. 2024;38(2):155–67. doi: 10.1038/s41371-023-00861-7 37857758 PMC10844095

[15] Tan M, He FJ, Wang C, MacGregor GA. Twenty-Four-Hour Urinary Sodium and Potassium Excretion in China: A Systematic Review and Meta-Analysis. J Am Heart Assoc. 2019;8(14):e012923. doi: 10.1161/JAHA.119.012923 31295409 PMC6662145

[16] Global burden of 288 causes of death and life expectancy decomposition in 204 countries and territories and 811 subnational locations, 1990-2021: a systematic analysis for the Global Burden of Disease Study 2021. Lancet. 2024;403(10440):2100–32. 38582094 10.1016/S0140-6736(24)00367-2 PMC11126520

[17] Global burden and strength of evidence for 88 risk factors in 204 countries and 811 subnational locations, 1990-2021: a systematic analysis for the Global Burden of Disease Study 2021. Lancet. 2024;403(10440):2162–203. 38762324 10.1016/S0140-6736(24)00933-4 PMC11120204

[18] Murray CJL. Findings from the Global Burden of Disease Study 2021. The Lancet. 2024;403(10440):2259–62. 10.1016/S0140-6736(24)00769-4 38762327

[19] Kim HJ, Fay MP, Feuer EJ, Midthune DN. Permutation tests for joinpoint regression with applications to cancer rates. Stat Med. 2000;19(3):335–51. doi: 10.1002/(sici)1097-0258(20000215)19:3<335::aid-sim336>3.0.co;2-z 10649300

[20] Zhan Z, Chen B, Teng W, Huang R, Zheng H, Zhou S, et al. Trends and projections of gallbladder and biliary tract cancer in China: a comprehensive analysis from 1990 to 2030 based on the Global Burden of Disease Study 2021. BMC Public Health. 2025;25(1):2409. doi: 10.1186/s12889-025-23601-7 40629282 PMC12235985

[21] Holford TR. Approaches to fitting age-period-cohort models with unequal intervals. Stat Med. 2006;25(6):977–93. doi: 10.1002/sim.2253 16143994

[22] Zhan Z, Zheng X, Xu S, Zheng H, Zheng L, Wang J, et al. Rising burden of pancreatic cancer in China: Trends, drivers, and future projections. PLoS One. 2025;20(7):e0327009. doi: 10.1371/journal.pone.0327009 40591568 PMC12212494

[23] Cheng X, Yang Y, Schwebel DC, Liu Z, Li L, Cheng P, et al. Population ageing and mortality during 1990-2017: A global decomposition analysis. PLoS medicine. 2020;17(6):e1003138. 10.1371/journal.pmed.1003138 PMC7279585 32511229

[24] Zhan Z, Chen B, Lin W, Chen X, Huang R, Yang C, et al. Rising Burden of Colon and Rectum Cancer in China: An Analysis of Trends, Gender Disparities, and Projections to 2030. Ann Surg Oncol. 2025;32(5):3361–71. doi: 10.1245/s10434-025-16905-w 39836276

[25] Stevens GA, Alkema L, Black RE, Boerma JT, Collins GS, Ezzati M, et al. Guidelines for Accurate and Transparent Health Estimates Reporting: the GATHER statement. Lancet. 2016;388(10062):e19–23. doi: 10.1016/S0140-6736(16)30388-9 27371184

[26] Sharma R. Burden of stomach cancer incidence, mortality, disability-adjusted life years, and risk factors in 204 countries, 1990–2019: An examination of global burden of disease 2019. Journal of Gastrointestinal Cancer. 2024;55(2):787–99. 38265570 10.1007/s12029-023-01005-3

[27] Yang P, Huang W, Xu Y, Teng Y, Shu P. Trends and projections of the burden of gastric cancer in China and G20 countries: a comparative study based on the global burden of disease database 2021. Int J Surg. 2025;111(7):4854–65. doi: 10.1097/JS9.0000000000002464 40359560

[28] Qin N, Fan Y, Yang T, Yang Z, Fan D. The burden of Gastric Cancer and possible risk factors from 1990 to 2021, and projections until 2035: findings from the Global Burden of Disease Study 2021. Biomark Res. 2025;13(1):5. doi: 10.1186/s40364-024-00720-8 39773334 PMC11708091

[29] Gaddy JA, Radin JN, Loh JT, Zhang F, Washington MK, Peek RM Jr, et al. High dietary salt intake exacerbates Helicobacter pylori-induced gastric carcinogenesis. Infect Immun. 2013;81(6):2258–67. doi: 10.1128/IAI.01271-12 23569116 PMC3676043

[30] Steck SE, Murphy EA. Dietary patterns and cancer risk. Nat Rev Cancer. 2020;20(2):125–38. doi: 10.1038/s41568-019-0227-4 31848467

[31] Fang X, Wei J, He X, An P, Wang H, Jiang L, et al. Landscape of dietary factors associated with risk of gastric cancer: A systematic review and dose-response meta-analysis of prospective cohort studies. Eur J Cancer. 2015;51(18):2820–32. doi: 10.1016/j.ejca.2015.09.010 26589974

[32] Hipgrave DB, Chang S, Li X, Wu Y. Salt and Sodium Intake in China. JAMA. 2016;315(7):703–5. doi: 10.1001/jama.2015.15816 26881375

[33] Wang TY. Lowering Dietary Sodium Intake-Implementing and Studying the Effectiveness of Public Health Interventions. JAMA Intern Med. 2020;180(6):887. doi: 10.1001/jamainternmed.2020.0909 32338702

[34] Wang Z, Butler LM, Wu AH, Koh W-P, Jin A, Wang R, et al. Reproductive factors, hormone use and gastric cancer risk: The Singapore Chinese Health Study. Int J Cancer. 2016;138(12):2837–45. doi: 10.1002/ijc.30024 26829904 PMC5912157

[35] Luan X, Niu P, Wang W, Zhao L, Zhang X, Zhao D, et al. Sex Disparity in Patients with Gastric Cancer: A Systematic Review and Meta-Analysis. J Oncol. 2022;2022:1269435. doi: 10.1155/2022/1269435 36385957 PMC9646304

[36] Yusefi AR, Bagheri Lankarani K, Bastani P, Radinmanesh M, Kavosi Z. Risk Factors for Gastric Cancer: A Systematic Review. Asian Pac J Cancer Prev. 2018;19(3):591–603. doi: 10.22034/APJCP.2018.19.3.591 29579788 PMC5980829

[37] Luo Y, Su B, Zheng X: Trends and Challenges for Population and Health During Population Aging - China, 2015-2050. China CDC weekly. 2021;3(28):593–8. 34594944 10.46234/ccdcw2021.158 PMC8393078

[38] Lobanov-Rostovsky S, He Q, Chen Y, Liu Y, Wu Y, Liu Y, et al. Growing old in China in socioeconomic and epidemiological context: systematic review of social care policy for older people. BMC Public Health. 2023;23(1):1272. doi: 10.1186/s12889-023-15583-1 37391766 PMC10311713

[39] Ren S, Cai P, Liu Y, Wang T, Zhang Y, Li Q, et al. Prevalence of Helicobacter pylori infection in China: A systematic review and meta-analysis. J Gastroenterol Hepatol. 2022;37(3):464–70. doi: 10.1111/jgh.15751 34862656

[40] Li M, Sun Y, Yang J, de Martel C, Charvat H, Clifford GM, et al. Time trends and other sources of variation in Helicobacter pylori infection in mainland China: A systematic review and meta-analysis. Helicobacter. 2020;25(5):e12729. doi: 10.1111/hel.12729 32686261

[41] Mozaffarian D, Fahimi S, Singh GM, Micha R, Khatibzadeh S, Engell RE, et al. Global sodium consumption and death from cardiovascular causes. N Engl J Med. 2014;371(7):624–34. doi: 10.1056/NEJMoa1304127 25119608

[42] Trieu K, Neal B, Hawkes C, Dunford E, Campbell N, Rodriguez-Fernandez R, et al. Salt Reduction Initiatives around the World - A Systematic Review of Progress towards the Global Target. PLoS One. 2015;10(7):e0130247. doi: 10.1371/journal.pone.0130247 26201031 PMC4511674

[43] Zhan Z, Chen X, Xu S, Li Q, Yu J, Guo Z, et al. Impact of high body mass index on gallbladder and biliary tract cancer burden in China: a comprehensive analysis of trends from 1990 to 2021. World J Surg Oncol. 2024;22(1):296. doi: 10.1186/s12957-024-03582-4 39529095 PMC11556143

[44] Zhan Z, Chen X, Zheng J, Xu J, Zhou S, Guo Z, et al. Burden of colon and rectum cancer attributable to processed meat consumption in China, 1990-2021. Frontiers in Nutrition. 2025;12:1488077. 40225336 10.3389/fnut.2025.1488077 PMC11985440

