# Supplementary material for: Impact of high sodium intake on stomach cancer burden in China: A comprehensive analysis from 1990 to 2021
Source: PLoS One. 2026 Jan 5;21(1):e0334593. doi: 10.1371/journal.pone.0334593 (PMC12768256; doi:10.1371/journal.pone.0334593)
Supplement: S1 Fig — Abbreviations: DALYs, disability-adjusted life years. (PDF) [file pone.0334593.s001.pdf]

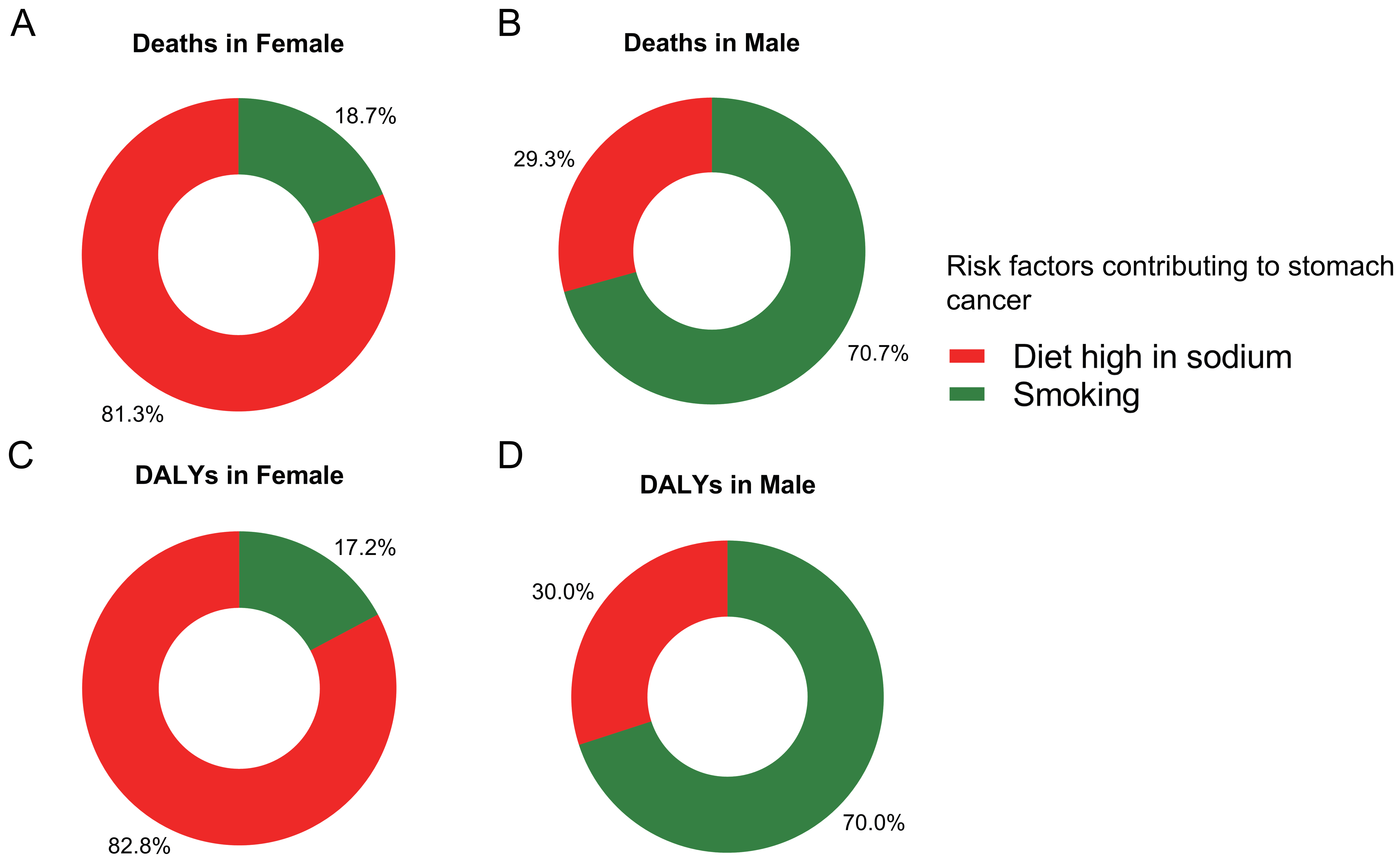
A
Deaths in Female
18.7%
81.3%
B
Deaths in Male
29.3%
70.7%
Risk factors contributing to stomach cancer
Diet high in sodium
Smoking
C
DALYs in Female
17.2%
82.8%
D
DALYs in Male
30.0%
70.0%
