# Supplementary material for: Impact of high sodium intake on stomach cancer burden in China: A comprehensive analysis from 1990 to 2021
Source: PLoS One. 2026 Jan 5;21(1):e0334593. doi: 10.1371/journal.pone.0334593 (PMC12768256; doi:10.1371/journal.pone.0334593)
Supplement: S1 Table — Abbreviations: DALYs, disability-adjusted life years. (DOCX) [file pone.0334593.s002.docx]

S1 Table. Trends in age-standardized mortality and DALY rates (per 100,000 persons) among both sexes, males, and females from 1990 to 2021 for stomach cancer attributable to diet high in sodium in China.

|  | Age-standardized mortality rate | | | Age-standardized DALY rate | | |
| --- | --- | --- | --- | --- | --- | --- |
| Gender | Period | APC (95% CI) | AAPC (95% CI) | Period | APC (95% CI) | AAPC (95% CI) |
| Both | 1990-1998 | -2.33 (-2.45 - -2.21) ^*^ | -2.45 (-2.61 - -2.30) ^*^ | 1990-1998 | -2.62 (-2.74 - -2.51) ^*^ | -2.76 (-2.91 - -2.61) ^*^ |
|  | 1998-2004 | 0.39 (0.15 - 0.63) ^*^ |  | 1998-2004 | -0.33 (-0.57 - -0.09) ^*^ |  |
|  | 2004-2007 | -6.20 (-7.21 - -5.17) ^*^ |  | 2004-2007 | -6.23 (-7.23 - -5.23) ^*^ |  |
|  | 2007-2010 | -2.79 (-3.84 - -1.71) ^*^ |  | 2007-2010 | -3.46 (-4.49 - -2.42) ^*^ |  |
|  | 2010-2015 | -4.16 (-4.49 - -3.83) ^*^ |  | 2010-2015 | -4.26 (-4.58 - -3.93) ^*^ |  |
|  | 2015-2021 | -1.91 (-2.09 - -1.73) ^*^ |  | 2015-2021 | -1.95 (-2.13 - -1.77) ^*^ |  |
| Female | 1990-1998 | -2.66 (-2.81 - -2.51) ^*^ | -2.91 (-3.12 - -2.70) ^*^ | 1990-1998 | -2.95 (-3.15 - -2.74) | -3.26 (-3.47 - -3.04) ^*^ |
|  | 1998-2004 | -0.49 (-0.81 - -0.17) ^*^ |  | 1998-2004 | -1.26 (-1.70 - -0.83) |  |
|  | 2004-2007 | -7.27 (-8.60 - -5.92) ^*^ |  | 2004-2007 | -7.20 (-9.02 - -5.35) |  |
|  | 2007-2010 | -3.97 (-5.36 - -2.56) ^*^ |  | 2007-2014 | -5.18 (-5.50 - -4.86) |  |
|  | 2010-2014 | -5.44 (-6.12 - -4.75) ^*^ |  | 2014-2021 | -1.62 (-1.88 - -1.35) |  |
|  | 2014-2021 | -1.41 (-1.61 - -1.21) ^*^ |  |  |  |  |
| Male | 1990-1998 | -2.20 (-2.36 - -2.04) ^*^ | -2.22 (-2.44 - -2.01) ^*^ | 1990-1998 | -2.49 (-2.63 - -2.35) ^*^ | -2.51 (-2.71 - -2.32) ^*^ |
|  | 1998-2004 | 0.96 (0.62 - 1.31) ^*^ |  | 1998-2004 | 0.16 (-0.15 - 0.47) |  |
|  | 2004-2007 | -5.67 (-7.08 - -4.24) ^*^ |  | 2004-2007 | -5.62 (-6.89 - -4.33) ^*^ |  |
|  | 2007-2010 | -2.23 (-3.70 - -0.74) ^*^ |  | 2007-2012 | -3.21 (-3.62 - -2.79) ^*^ |  |
|  | 2010-2015 | -3.92 (-4.37 - -3.46) ^*^ |  | 2012-2015 | -4.33 (-5.62 - -3.02) ^*^ |  |
|  | 2015-2021 | -2.20 (-2.45 - -1.94) ^*^ |  | 2015-2021 | -2.11 (-2.34 - -1.88) ^*^ |  |

Abbreviations: DALYs, disability-adjusted life-years; AAPC, average annual percent change presented for full period; APC, annual percent change; CI, confidence interval. ^*^, *p* <0.05.
