# Supplementary material for: Impact of high sodium intake on stomach cancer burden in China: A comprehensive analysis from 1990 to 2021
Source: PLoS One. 2026 Jan 5;21(1):e0334593. doi: 10.1371/journal.pone.0334593 (PMC12768256; doi:10.1371/journal.pone.0334593)
Supplement: S2 Table — Abbreviations: YLD, year lived with disability; YLL, year of life lost. (DOCX) [file pone.0334593.s003.docx]

S2 Table. Trends in age-standardized YLD, and YLL rates (per 100,000 persons) among both sexes, males, and females from 1990 to 2021 for stomach cancer attributable to diet high in sodium in China.

|  | Age-standardized YLD rate | | | Age-standardized YLL rate | | |
| --- | --- | --- | --- | --- | --- | --- |
| Gender | Period | APC (95% CI) | AAPC (95% CI) | Period | APC (95% CI) | AAPC (95% CI) |
| Both | 1990-1998 | -1.67 (-1.79 - -1.55) ^*^ | -1.27 (-1.43 - -1.12) ^*^ | 1990-1998 | -2.63 (-2.75 - -2.52) ^*^ | -2.77 (-2.93 - -2.62) ^*^ |
|  | 1998-2004 | 1.31 (1.06 - 1.56) ^*^ |  | 1998-2004 | -0.35 (-0.59 - -0.11) ^*^ |  |
|  | 2004-2007 | -3.93 (-4.97 - -2.88) ^*^ |  | 2004-2007 | -6.26 (-7.26 - -5.25) ^*^ |  |
|  | 2007-2010 | -1.41 (-2.47 - -0.33) ^*^ |  | 2007-2010 | -3.49 (-4.52 - -2.44) ^*^ |  |
|  | 2010-2015 | -2.66 (-2.99 - -2.32) ^*^ |  | 2010-2015 | -4.28 (-4.60 - -3.95) ^*^ |  |
|  | 2015-2021 | -0.72 (-0.91 - -0.54) ^*^ |  | 2015-2021 | -1.96 (-2.15 - -1.78) ^*^ |  |
| Female | 1990-1998 | -2.09 (-2.30 - -1.89) ^*^ | -1.91 (-2.12 - -1.70) ^*^ | 1990-1998 | -2.96 (-3.16 - -2.75) ^*^ | -3.27 (-3.49 - -3.06) ^*^ |
|  | 1998-2004 | 0.15 (-0.29 - 0.60) |  | 1998-2004 | -1.28 (-1.71 - -0.84) ^*^ |  |
|  | 2004-2007 | -5.18 (-6.99 - -3.34) ^*^ |  | 2004-2007 | -7.23 (-9.04 - -5.38) ^*^ |  |
|  | 2007-2015 | -3.27 (-3.52 - -3.01) ^*^ |  | 2007-2014 | -5.20 (-5.52 - -4.88) ^*^ |  |
|  | 2015-2021 | -0.21 (-0.54 - 0.13) |  | 2014-2021 | -1.63 (-1.90 - -1.36) ^*^ |  |
|  |  |  |  |  |  |  |
| Male | 1990-1998 | -1.52 (-1.67 - -1.38) ^*^ | -1.00 (-1.20 - -0.80)  ^*^ | 1990-1998 | -2.50 (-2.64 - -2.36) ^*^ | -2.53 (-2.73 - -2.34) ^*^ |
|  | 1998-2004 | 1.87 (1.55 - 2.18) ^*^ |  | 1998-2004 | 0.14 (-0.17 - 0.46) |  |
|  | 2004-2007 | -3.27 (-4.59 - -1.94) ^*^ |  | 2004-2007 | -5.64 (-6.92 - -4.35) ^*^ |  |
|  | 2007-2012 | -1.28 (-1.70 - -0.85) ^*^ |  | 2007-2012 | -3.23 (-3.65 - -2.82) ^*^ |  |
|  | 2012-2015 | -2.89 (-4.20 - -1.55) ^*^ |  | 2012-2015 | -4.35 (-5.65 - -3.04) ^*^ |  |
|  | 2015-2021 | -0.79 (-1.02 - -0.56) ^*^ |  | 2015-2021 | -2.13 (-2.37 - -1.90) ^*^ |  |

Abbreviations: YLDs, years lived with disability; YLLs, years of life lost; AAPC, average annual percent change presented for full period; APC, annual percent change; CI, confidence interval. ^*^, *p* <0.05.
