# Supplementary figures and images for: Impact of high sodium intake on stomach cancer burden in China: A comprehensive analysis from 1990 to 2021
Source: PLoS One. 2026 Jan 5;21(1):e0334593. doi: 10.1371/journal.pone.0334593 (PMC12768256; doi:10.1371/journal.pone.0334593)

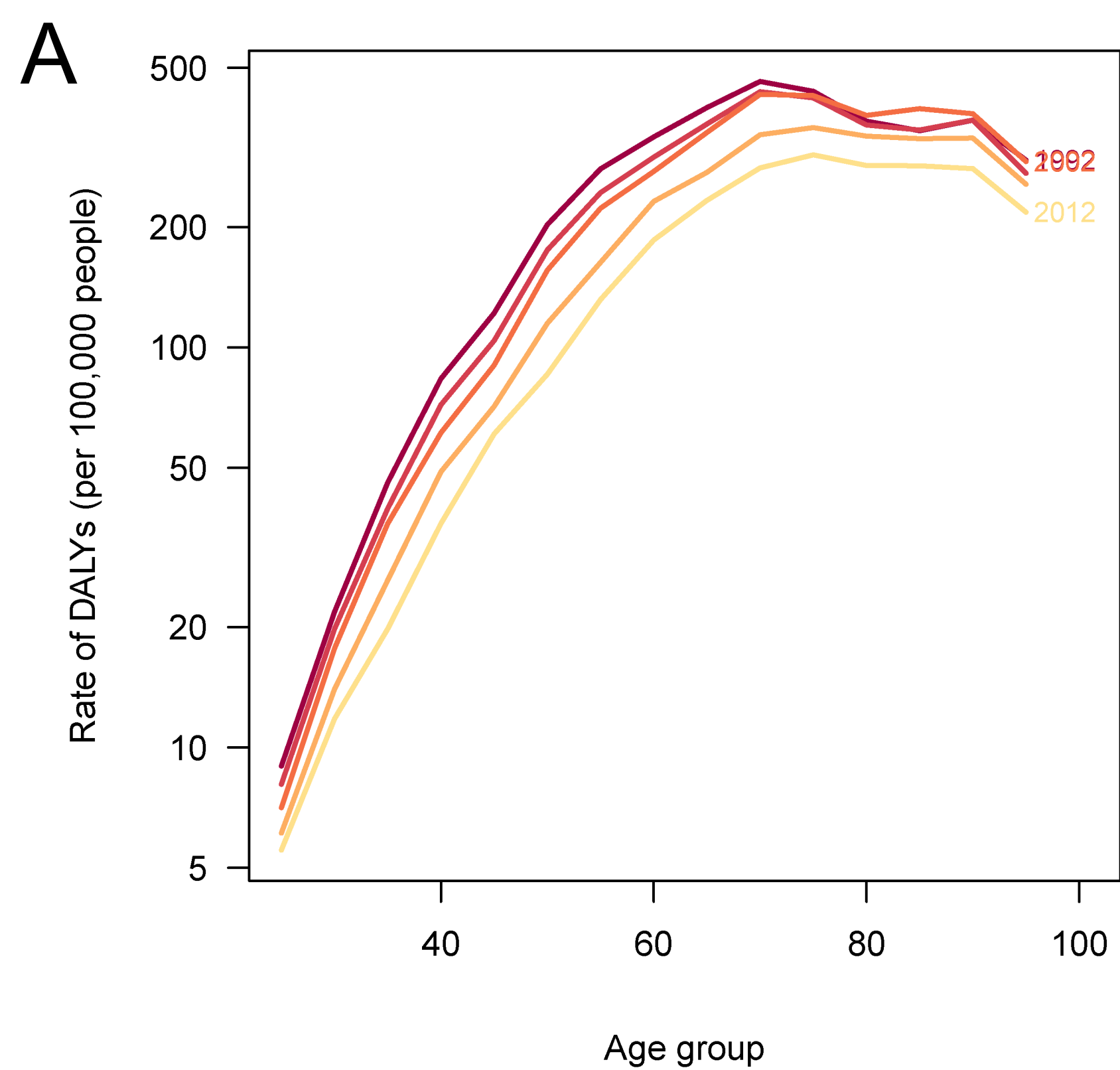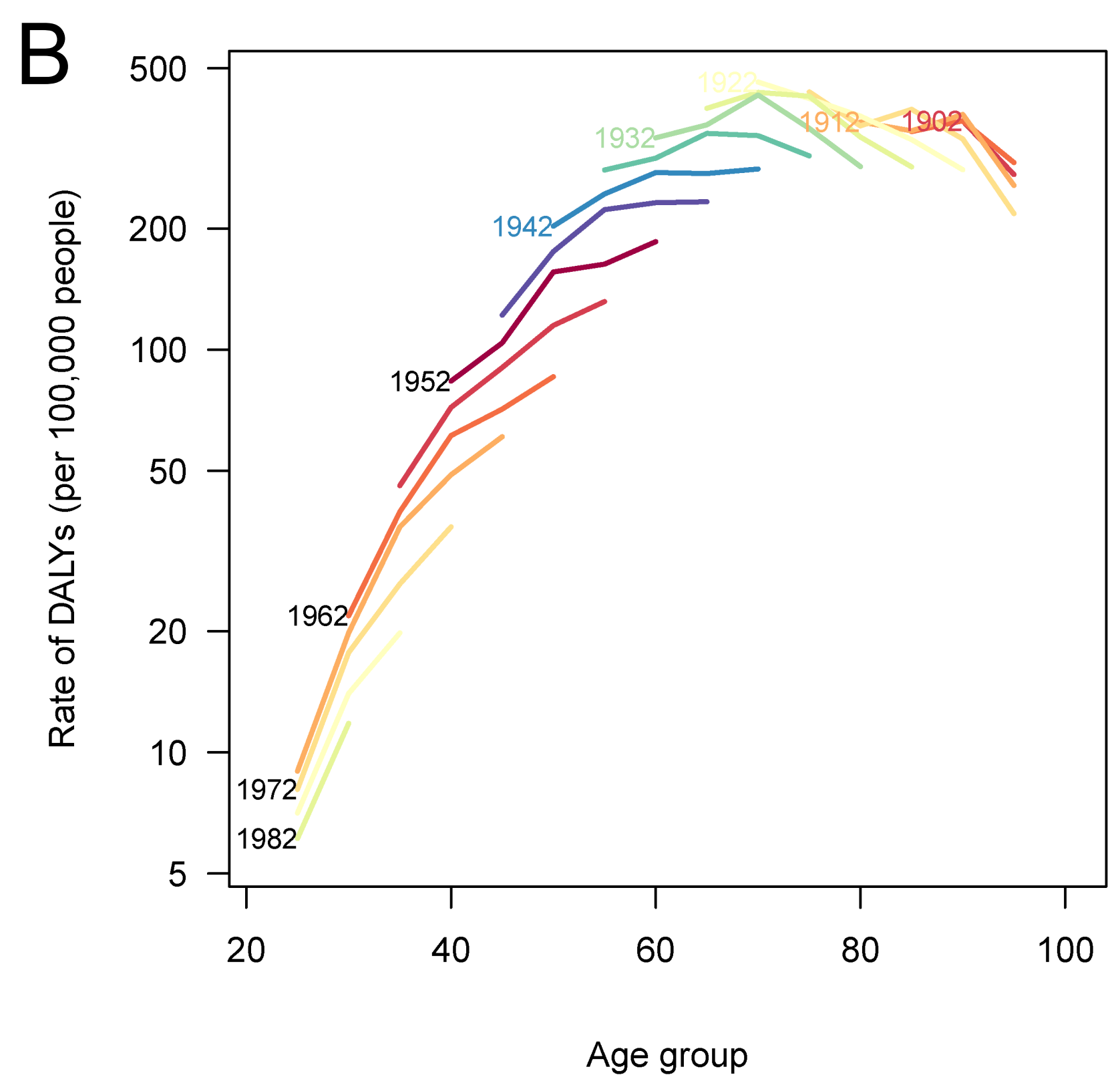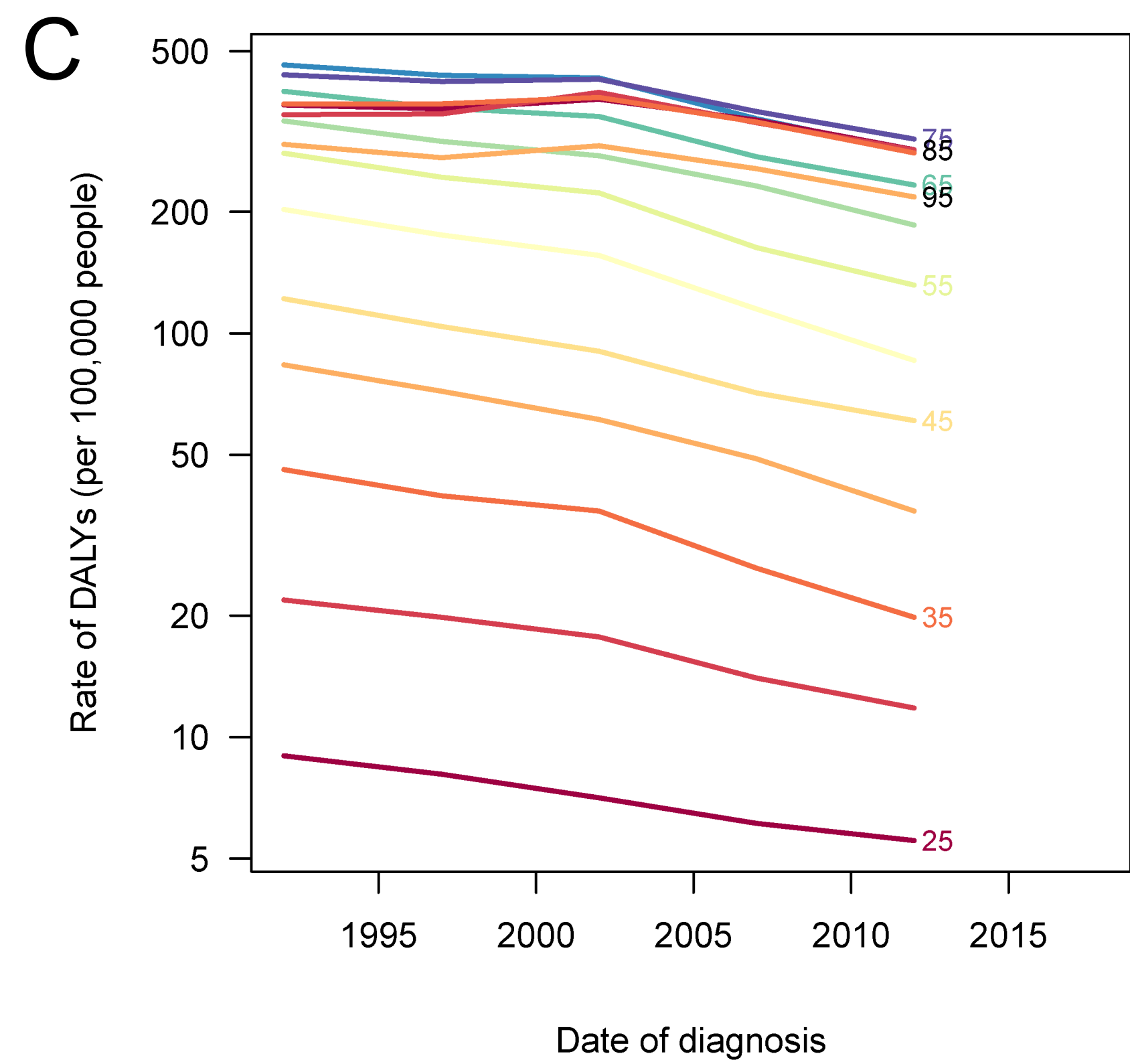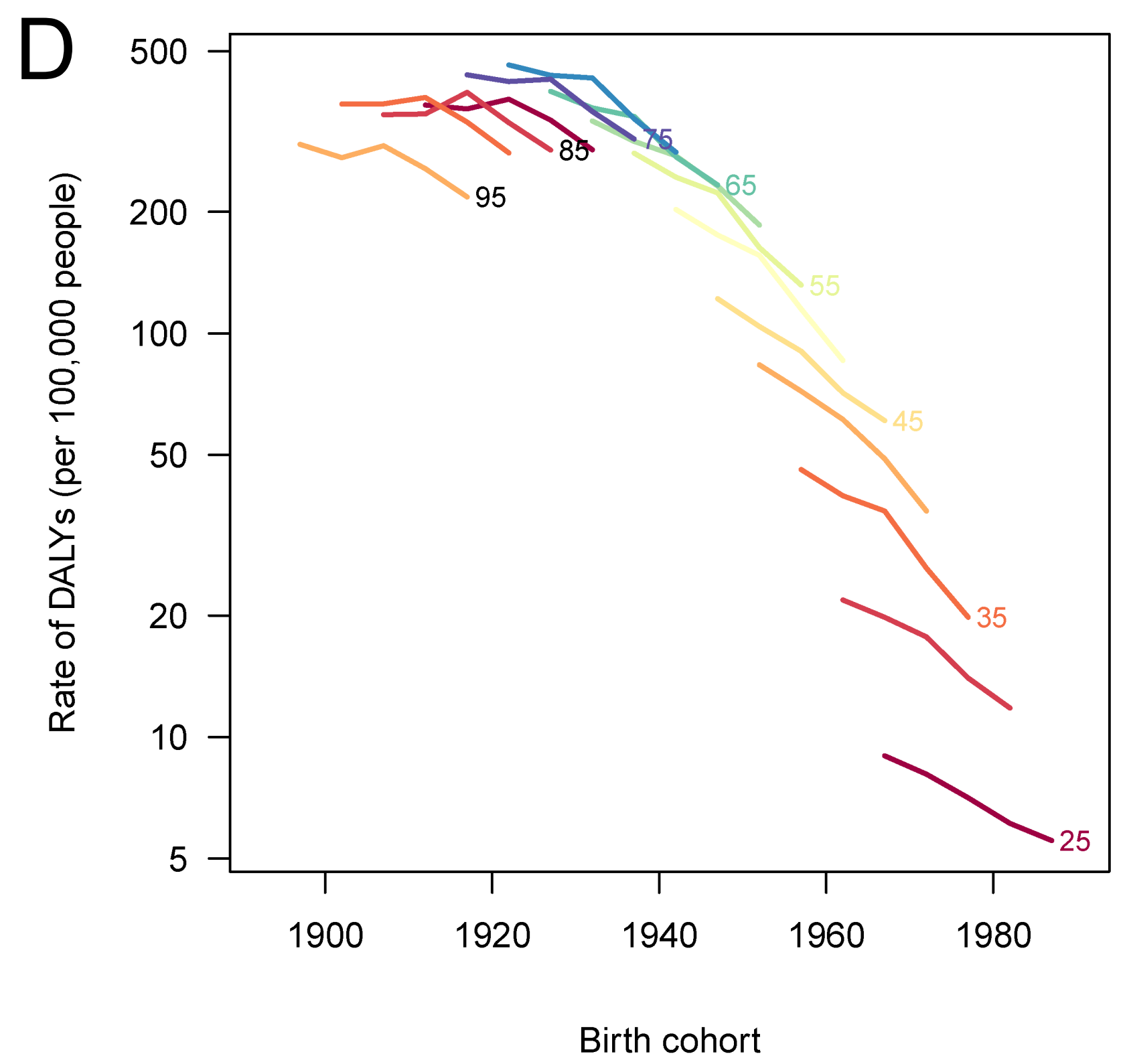

Supplement: S2 Fig — (A) The age-specific DALYs rates according to time periods; each line connects the age-specific rates for a 5-year period. (B) The age-specific DALYs rates according to birth cohorts; each line connects the age-specific rates for a 5-year birth cohort. (C) The period-specific DALYs rates according to age groups; each line connects the period-specific rates for a 5-year age group. (D) The cohort-specific DALYs rates according to age groups; each line connects the cohort-specific rates for a 5-year age group. Abbreviations: DALYs, disability-adjusted life years. (PDF) [file pone.0334593.s004.pdf]
